# CTSB Nuclear Translocation Facilitates DNA Damage and Lysosomal Stress to Promote Retinoblastoma Cell Death

**DOI:** 10.1007/s12033-023-01042-0

**Published:** 2023-12-30

**Authors:** Cairui Li, Shuguang Sun, Yanmei Zhuang, Zhaokui Luo, Guangquan Ji, Zhong Liu

**Affiliations:** 1 Department of Ophthalmology, Dali Prefecture People’s Hospital (The Third Affiliated Hospital of Dali University), Dali, Yunnan 671003 China; 2 Department of Endocrine, The First Affiliated Hospital of Dali University, Dali, Yunnan 671003 China; 3 Department of Ophthalmology, Weishan County People’s Hospital, Weishan, Yunnan 672400 China; 4 Department of Ophthalmology, Jingdong County Hospital in Yunnan Province, Jingdong, Yunnan 665700 China; 5 Department of Ophthalmology, Jingdong County Traditional Chinese Medicine Hospital in Yunnan Province, Jingdong, Yunnan 665700 China; 6 Department of Surgery, Weishan County People’s Hospital, Weishan, Yunnan 672400 China

**Keywords:** Retinoblastoma, CTSB nuclear translocation, DNA damage repair, Lysosomal stress, Ferroptosis, Autophagy

## Abstract

Retinoblastoma (RB) is a pernicious tumor originating from photoreceptor precursor cells that often endangers the lives of children. The purpose of our study was to further investigate the influence of cathepsin B (CTSB) nuclear translocation on RB cell death. Y79 cells were injected into the vitreous cavity of nude mice at a dose of 4 µL/mouse to establish an animal model of RB. Real-time quantitative polymerase chain reaction (RT–qPCR), Western blot analysis, a comet assay, a Cell Counting Kit-8 (CCK-8) assay and flow cytometry were used to measure the levels of the interrelated genes and proteins and to evaluate alterations in autophagy, apoptosis, proliferation, DNA damage and cell cycle arrest. CTSB was found to be expressed at low levels in RB animal model samples and RB cell lines. Functionally, CTSB nuclear translocation promoted DNA damage, cell cycle arrest, ferroptosis and autophagy in Y79 cells and inhibited their proliferation. Downstream mechanistic studies showed that nuclear translocation of CTSB facilitates DNA damage and cell cycle arrest in RB cells by inhibiting breast cancer 1 protein (BRCA1) expression and also activates the signal transducer and activator of transcription 3/stimulator of interferon response cGAMP interactor 1 (STAT3/STING1) pathway to induce lysosomal stress, leading to ferroptosis and autophagy in Y79 cells and alleviating RB. Nuclear translocation of CTSB facilitates DNA damage and cell cycle arrest in RB cells by inhibiting BRCA1 expression and activating the STAT3/STING1 pathway and induces lysosomal stress, which eventually leads to ferroptosis and autophagy and mitigates RB.

**Supplementary Information:**

The online version contains supplementary material available at 10.1007/s12033-023-01042-0.

## Introduction

Retinoblastoma (RB) is the most common childhood intraocular malignancy and is lethal if left untreated [1]. The two most common symptoms of RB are leukocoria and strabismus [2]. RB is reported in 1 in 16,000 births and is diagnosed in 8,000–10,000 children each year [3]. RB can be classified as hereditary and nonhereditary. In hereditary RB, germline mutations in the RB1 gene predispose individuals to RB development, with only one additional somatic mutation in the remaining RB1 allele required to cause tumor formation, while in nonhereditary RB, two independent somatic mutations or epigenetic silencing through methylation are required [4]. The prognosis of RB in low- and middle-income countries is very poor, and more than 80% of RB cases worldwide occur in low- and middle-income countries [5, 6]. Therefore, the development of new strategies for treating RB is urgently needed.

Cathepsin B (CTSB) is a cathepsin belonging to the cysteine cathepsin family, whose members are responsible for proteolytic degradation in lysosomes and lysosomes in vitro environments, where they play an integral roles in autophagy, antigen presentation, cellular stress signaling, metabolism, and lysosome-dependent cell death [7]. The proapoptotic influence of CTSB, especially the active enzymatic domain of CTSB, is related to inhibition of breast cancer 1 (BRCA1) protein activity in the nucleus [8]. BRCA1 is a tumor suppressor expressed in all cells and is involved in essential functions required for cell replication and DNA synthesis [9]. Several studies have shown that CTSB can exert its proteolytic effect either intracellularly or extracellularly, depending on the cell type and its localization [10, 11]. Wang [8] showed that RD-N (an aminomethylated derivative of riccardin D) induces CTSB translocation from lysosomes to the nucleus, and CTSB induces DNA damage in the nucleus and mediates BRCA1 degradation, resulting in DNA repair defects. Nagakannan [12] showed that lysosomal membrane penetration and cytoplasmic spillover of CTSB are the main causes of ferroptosis. Man [7] showed that CTSB may restrain lysosomal biogenesis and autophagy under steady-state conditions. However, whether nuclear translocation of CTSB affects the expression of BRCA1 and involved in the RB process remains unknown. This study explored the molecular mechanism by which CTSB affects RB-related processes.

DNA, the basic unit of heredity, is an intrinsically reactive molecule that is highly susceptible to chemical modification by both endogenous and exogenous substances [13]. DNA damage can cause cell death or senescence, thus impairing the function of metabolic organs, and can also cause tissue inflammation and disrupt the systemic metabolic homeostasis [14]. DNA repair is an important defense mechanism against DNA damage and involves 4 distinct pathways: mismatch, excision, direct and double-strand break repair. It has been reported that DNA damage repair plays an important role in RB occurrence and progression [15]. Carnevale [16] reported that DNA damage signaling induces apoptosis via distinctively modified E2F transcription factor 1 (E2F1) proteins. Therefore, the regulation of DNA damage repair can regulate apoptosis, and this study speculated that CTSB may play a role in regulating tumorigenesis in RB by influencing DNA damage repair.

Lysosomes are components of the macrolysosomal system, which also includes endosomes, multivesicular bodies (MVBs), autophagosomes, and autophagolysosomes. Lysosomes play an important role in the pathogenesis of multifarious diseases, including neurodegenerative diseases and cancer [17]. It has been reported that endoplasmic reticulum-associated oxidative stress, lysosomal dysfunction, and Golgi stress-associated lipid peroxidation are conducive to the induction of ferroptosis [18]. Likewise, lysosomes play an integral role in distinct types of autophagy, including microautophagy, macroautophagy, and chaperone-mediated autophagy, as well as in pathways mediating multiple forms of cell death, such as lysosomal cell death, apoptotic cell death, and autophagic cell death [19]. Lu [20] showed that 3-chloro-1,2-propanediol (3-MCPD) can block autophagic flux by suppressing lysosomal function, thus inducing liver injury. Here, this study speculated that CTSB may play a role in RB by influencing lysosomal stress, thereby promoting ferroptosis and autophagy.

Therefore, to address the above hypotheses, this study explored the potential function and molecular mechanism of CTSB nuclear translocation in RB development and investigated whether CTSB nuclear translocation plays a role in the occurrence and development of RB by affecting DNA damage repair and lysosomal stress to provide a potential theoretical basis for the treatment of RB.

## Materials and Methods

### Materials

RPMI 1640 medium, DMEM/F12, fetal bovine serum (FBS), and penicillin/streptomycin were purchased from HyClone Laboratories, Inc. (Logan, USA). Radioimmunoprecipitation assay (RIPA) lysis buffer, TRIzol reagent, a First Strand cDNA Synthesis Kit, a SYBR Green real-time quantitative polymerase chain reaction (RT‒qPCR) kit, a bicinchoninic acid (BCA) kit, an enzyme-linked immunosorbent assay (ELISA) kit, and a malondialdehyde (MDA) kit were purchased from Solarbio Science & Technology Co., Ltd. (Beijing, China). Cell Counting Kit-8 (CCK-8) reagent, a coimmunoprecipitation (Co-IP) kit and a reactive oxygen species (ROS) assay kit were obtained from Beyotime Technologies (Shanghai, China). An Annexin V-fluorescein isothiocyanate/propidium iodide (Annexin V-FITC/PI) apoptosis kit and a comet assay kit were purchased from Keygen Biotech Co., Ltd. (Nanjing, China). The antibodies used in this study were purchased from Abcam (Cambridge, UK).

### Animal Model Establishment and Grouping

Thirty male BALB/c nude mice (weight: 16–18 g, age: 4–6 weeks) were obtained from Hunan SJA Laboratory Animal Co., Ltd. After 2 weeks of adaptive feeding, the mice were randomly divided into 3 groups: the RB group, OE-CTSB group and si-CTSB group (10 nude mice in each group). Four microliters of a suspension of Y79 cells (3.5 × 10^7^ cells/mL) or Y79 cells stably transfected with OE-CTSB or si-CTSB was aspirated, and the needle was inserted into the pars plana of the ciliary body of nude mice under microscopic visualization, with the needle tip facing the optic disc; a sensation of emptiness was felt when the needle entered the vitreous cavity. The cell suspension was injected into the vitreous cavity, and the needle was slowly withdrawn. Ocular erythromycin ointment was applied to both eyes after the operation. After 6 weeks [21], the mice were killed, and retinal tissues were harvested for follow-up experiments.

### Cell Culture

The human retinal pigment epithelial cell line ARPE-19 and the retinoblastoma cell lines Y79 and SO-RB50 were obtained from Otwo Biotechnology Co., Ltd. (Shenzhen, China). Y79 and SO-RB50 cells were cultured in RPMI 1640 medium, and ARPE-19 cells were cultured in a 1:1 mixture of Dulbecco’s modified Eagle’s medium and Ham’s F-12 (DMEM/F12) medium. The media used were supplemented with 10% FBS and 1% penicillin/streptomycin, and cells were incubated at 37 °C with 5% CO_2_. When the cells reached 80% confluency, they were passaged, and experiments were carried out on cells in the logarithmic growth phase.

### Cell Transfection

The OE-CTSB, si-CTSB, OE-BRCA1, and si-STAT3 constructs were synthesized by RiboBio (Guangzhou, China). Y79 cells were cultured overnight in 24-well plates, and when the cells were approximately 60 to 70% confluent, they were transfected with OE-CTSB, si-CTSB, OE-BRCA1, or si-STAT3 using Lipofectamine 3000 reagent (Invitrogen, Waltham, USA) according to the manufacturer’s instructions and cultured for 48 h at 37 °C in a 5% CO_2_ incubator. Then, the transfection efficiency was evaluated, and the cells were used for follow-up experiments.

### RT‒qPCR

Total RNA was extracted from tissues and cells with TRIzol Reagent, and cDNA was synthesized by reverse transcription of 1 µg of RNA with a First-Strand cDNA Synthesis Kit. RT‒qPCR was performed using a SYBR Green RT‒qPCR Kit, with glyceraldehyde 3-phosphate dehydrogenase (GAPDH) serving as the internal reference. The thermal cycling conditions for PCR were as follows: 40 cycles of predenaturation at 95 °C for 20 s, denaturation at 95 °C for 10 s, and annealing/extension at 60 °C for 20 s. The detailed primer sequences are shown in Table 1, and relative expression levels were calculated by the 2^−ΔΔCt^ method [22].

**Table 1:** Primer sequences

Target	Sequence (5´-3´)
CTSB	F: CAGACCGTACTCCATCCC R: GTGCCATTCTCCACTCCC
LAMP1	F: TGACAAGGCTTCTCAACATC R: CCAGCAGACACTCCTCCAC
GBA	F: AGCGTCCCGTTTCACTCC
	R: GCAGCAAGCGTTGGTCAT
GNS	F: GCAAGCCAAGACTCCAAT
	R: TTAGGTCGTAGCCAGCAA
GAPDH	F: GGGAAACTGTGGCGTGAT
	R: AAAGGTGGAGGAGTGGGT

F: Forward; R: Reverse; LAMP1: Lysosome-associated membrane protein 1; GBA: Glucocerebrosidase; GNS: N-acetylglucosamine-6-sulfatase

### Western Blot Analysis

Western blot analysis was performed according to a previous description [23]. Total protein was extracted from cells and retinal tissue with RIPA buffer supplemented with 1% protease inhibitor solution. The protein concentration was determined according to the instructions of the BCA Protein Assay Kit. Total protein (30 µg/lane) was separated by SDS‒PAGE, and the separated proteins were subsequently transferred to a polyvinylidene fluoride (PVDF) membrane (Merck Millipore, MA, USA), which was then blocked with 5% skim milk powder at room temperature for 1.5 h. Diluted primary antibodies specific for the following proteins were added: microtubule-associated protein 1 light chain 3 (LC3I/II, 1:1000, ab128025), Beclin1 (1:2000, ab207612), p62 (1:1000, ab109021), CTSB (1:1000, ab214428), ataxia-telangiectasia mutated kinase (ATM, 1:1000, ab32420), phosphorylated ATM (p-ATM, 1:5000, ab81292), checkpoint kinase 2 (Chk2, 1:1000,ab109413), phosphorylated-Chk2 (p-Chk2, 1:1000, ab278548), gamma-phosphorylated H2A histone family member X (γH2AX, 1:1000, ab81299), BRCA1 (1: 1000, ab191042), p-BRCA1 (1:1000, ab90528), P18 (1:1000, ab192239), P21 (1:1000, ab109520), Cyclin D1 (1:2000, ab16663), signal transducer and activator of transcription 3 (STAT3, 1:1000, ab68153), phosphorylated-STAT3 (p-STAT3, 1:1000, ab267373), stimulator of interferon response cGAMP interactor 1 (STING1, 1:1000, ab239074), glutathione peroxidase 4 (GPX4, 1:1000, ab125066), ferritin heavy chain 1 (FTH1, 1:1000, ab266581), solute carrier family 7 member 11 (SLC7A11, 1:10000, ab175186), heme oxygenase-1 (HO-1, 1:1000, ab305290), B-cell lymphoma 2 (Bcl-2, 1:500, ab194583), Caspase-3 (1:5000, ab214430). The membrane was then incubated overnight at 4 °C. Next, the membrane was incubated with the corresponding secondary antibody (1:2000, ab97051) for 1 h at room temperature, and bands were visualized with an enhanced chemiluminescence (ECL) kit. Finally, relative quantification of band densities was carried out with ImageJ software.

### Evaluation of Cell Proliferation by a CCK-8 Assay

In this study, a CCK-8 kit was used to detect cell proliferation activity [24]. Y79 cells (5 × 10^3^ cells/well) were seeded in 96-well plates and cultured at 37 °C in a 5% CO_2_ incubator for 24 h. The cells in each group were treated and cultured for another 24 h, after which 10 µL of CCK-8 reagent was added to each well. The absorbance at 450 nm was subsequently measured with an enzyme labeler after incubation for 2 h.

### Detection of Apoptosis by Flow Cytometry

Apoptosis was detected using an Annexin V-FITC/PI kit as previously described [22]. Y79 cells from each group were collected, washed twice with PBS, and then suspended in 200 µL of PBS. Five microliters of Annexin V-FITC and 5 µL of PI were added, and the cells were incubated for 15 min in the dark, after which apoptosis was detected with a FACScan flow cytometer.

### Measurement of ROS by Flow Cytometry

As described previously [25], an ROS assay kit was used to measure the content of ROS. Y79 cells were collected after trypsin digestion, the cell concentration was adjusted with cell culture medium, and the cells were seeded into a 6-well plate (2 × 10^5^ cells/well) and cultured in a cell incubator containing 5% CO_2_ for 24 h. After centrifugation, the medium was discarded, and the cells were suspended in PBS. Then, 10 µL of DCFH-DA was added, and the cells were incubated in a cell incubator for 30 min in the dark. To ensure complete binding of the probe to the cells, the cells were shaken every 5 min during this period. Then, the cells were centrifuged at 1500 × g for 5 min and washed twice with PBS. PBS was then added again, and the ROS content in the cells was quantified via flow cytometry.

### Analysis of the Cell Cycle Distribution by Flow Cytometry

As described in previous research [26], when the confluence of Y79 cells reached 80%, the cells were digested with trypsin and centrifuged at 1200 rpm for 5 min, the supernatant was discarded, the cells were fixed with 70% precooled ethanol overnight at 4 °C, and a specific volume of propidium iodide (PI) staining solution (1-1.5 ml) was added according to the number of cells. The cell cycle distribution was then evaluated using flow cytometry at a flow rate of 200–350 cells/s.

### Comet Assay

The comet assay was performed using a DNA damage detection kit (KeyGEN, Nanjing, China). Y79 cells were harvested and suspended in PBS containing 1% low-melting-point agarose and were then layered onto a sticky microscope slide precoated with 0.5% normal-melting-point agarose. The slides were immersed in a specific lysis buffer at 4 °C for 2 h. The DNA was subsequently unwound in alkaline electrophoresis buffer for 30 min. DNA was stained with 50 µL of 30 µg/mL ethidium bromide (EB) for 20 min in the dark after electrophoresis. Finally, a fluorescence microscope (Nikon, Tokyo, Japan) was used to observe and photograph the cells. Comet analysis was performed as described previously [27].

### ELISA Test

Nude mouse retinal tissue and cell culture supernatants were collected after treatment following the instructions of the ELISA kit. The concentrations of the inflammatory cytokines interleukin-6 (IL-6), tumor necrosis factor alpha (TNF-α) and interleukin-1beta (IL-1β) were determined by measuring the optical density at 450 nm.

### Detection of MDA with a Kit

According to the instructions of the MDA kit (BC0025, Solarbio, Beijing, China), Y79 cells were collected into a centrifuge tube, the supernatant was discarded after centrifugation, and 1 mL of extraction buffer was added to every 5 million cells. The cells were lysed by ultrasonication (power, 200 W; ultrasonication, 3 s; interval, 10 s; repeated 30 times) and centrifuged at 8000 × g for 10 min. The supernatant was subsequently removed and placed on ice for testing. Finally, the samples were added step by step according to the instructions, and the absorbance at 532 and 600 nm was measured with a microplate reader. The experimental procedure was described in a previous article [28].

### Coimmunoprecipitation

Immunoprecipitation was performed using the Protein A + G magnetic bead method [29]. Y79 cells were harvested and lysed in 300 µL of lysis buffer. The cells were lysed by ultrasonication at 100 W for 5 s, after which the supernatant was obtained by centrifugation. Three micrograms of an anti-CTSB antibody or anti-BRCA1 antibody was added to the supernatant, which was subsequently incubated at 4 °C overnight. Then, the protein lysate and protein A/G magnetic beads were incubated at 4 °C overnight with gentle shaking. The beads were collected by centrifugation and washed 3 times with lysis buffer. The immunoprecipitates were analyzed by western blotting.

### Bioinformatics Analysis

#### Data Source

In this study, datasets related to RB were retrieved from the Gene Expression Omnibus (GEO) database, and the sample sources and processing methods were screened to reduce the effects of differences among the datasets. The GSE97508 dataset met the needs of this study; thus, it was selected for research on the molecular mechanism.

#### Analysis

Background checking and normalization were carried out on the mRNA data through the Limma package in R software, and further differential expression analysis was carried out. The threshold criterion for differential expression was *P* < 0.05, and log2FC values were filtered considering the threshold value for statistical significance in the dataset. Gene Ontology (GO) term and Kyoto Encyclopedia of Genes and Genomes (KEGG) pathway enrichment analyses were performed with the differentially expressed genes, and gene set enrichment analysis (GSEA) was performed to further evaluate the distribution trend in the table of genes ranked by the correlations between genes and phenotypes.

### Statistical Analysis

All the statistical analyses were performed using GraphPad Prism version 8.0 software. All the experiments were repeated at least 3 times. The data are expressed as the means ± standard deviations (SDs). Student’s *t* test was used to analyze the significance of differences between two groups, and one-way ANOVA was used to analyze the significance of differences among three or more groups. The statistical significance was established when p-value < 0.05.

## Results

### The Expression of CTSB in Retinoblastoma

**Fig. 1:**
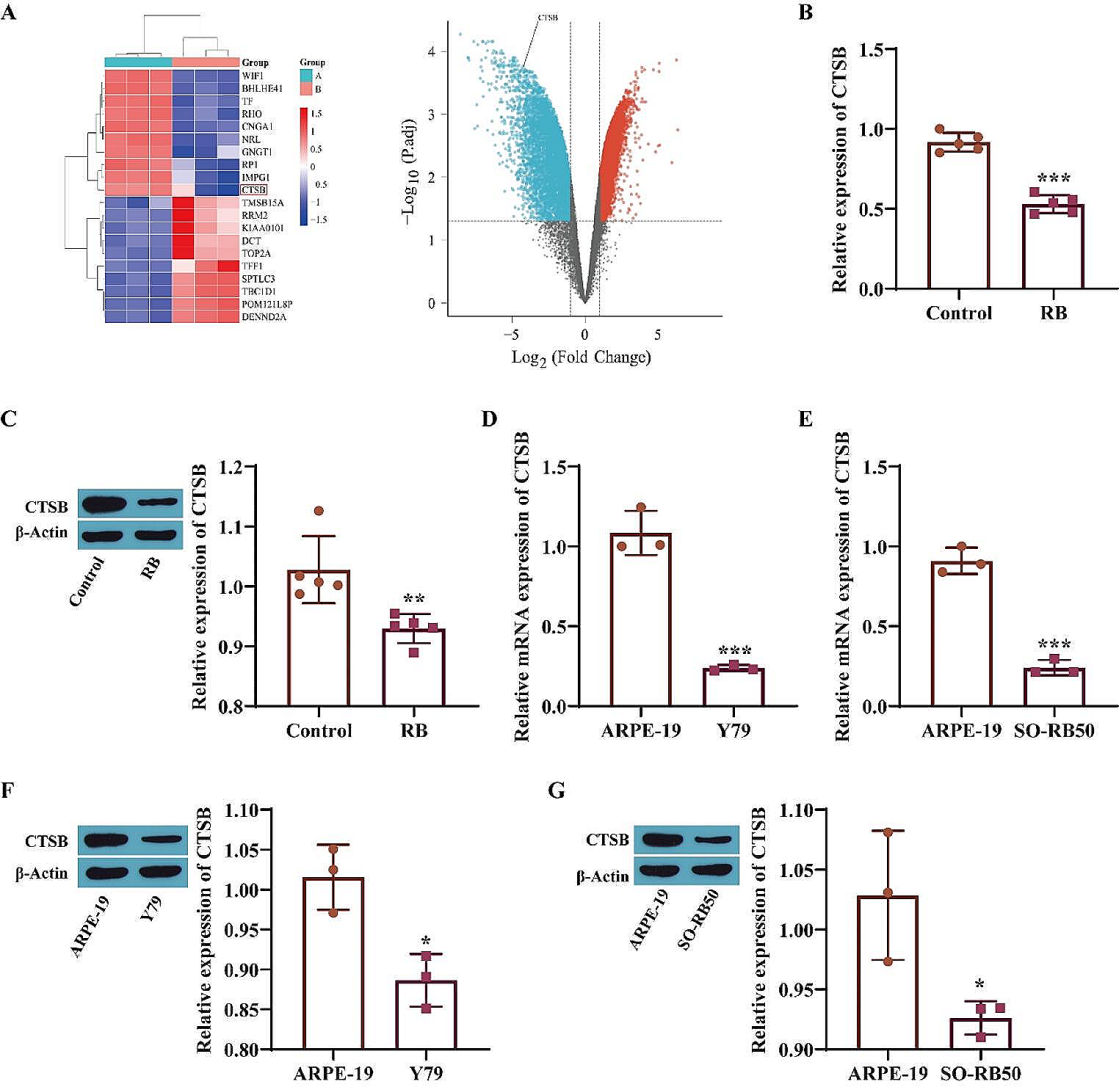
Expression of CTSB in retinoblastoma. **A**: The GSE97508 dataset was used to analyze differential gene expression. Group A: Normal group; Group B: RB group; **B**: RT‒qPCR was performed to measure CTSB levels; **C**: CTSB expression was evaluated via Western blot analysis; **D**‒**E**: CTSB expression was measured via RT‒qPCR; F‒G: CTSB protein levels were measured via Western blotting. *P < 0.05, **P < 0.01, ***P < 0.001 vs. the Control or ARPE-19 group

The GSE97508 dataset showed that CTSB was significantly downregulated in RB (Fig. 1A). Next, the expression of CTSB in RB was evaluated at the animal and cellular levels. The RT‒qPCR and Western blot results revealed that the CTSB level in the RB group was markedly lower than that in the control group (Fig. 1B-C). In addition, the expression of CTSB in ARPE-19, Y79 and SO-RB50 cells was measured, and the results showed that the expression level of CTSB in Y79 and SO-RB50 cells was significantly lower than that in ARPE-19 cells (Fig. 1D-G); thus, Y79 cells were selected for subsequent experiments. These results indicate that CTSB is expressed at low levels in RB.

### CTSB Induces Autophagy-Dependent Death in Retinoblastoma Cells

**Fig. 2:**
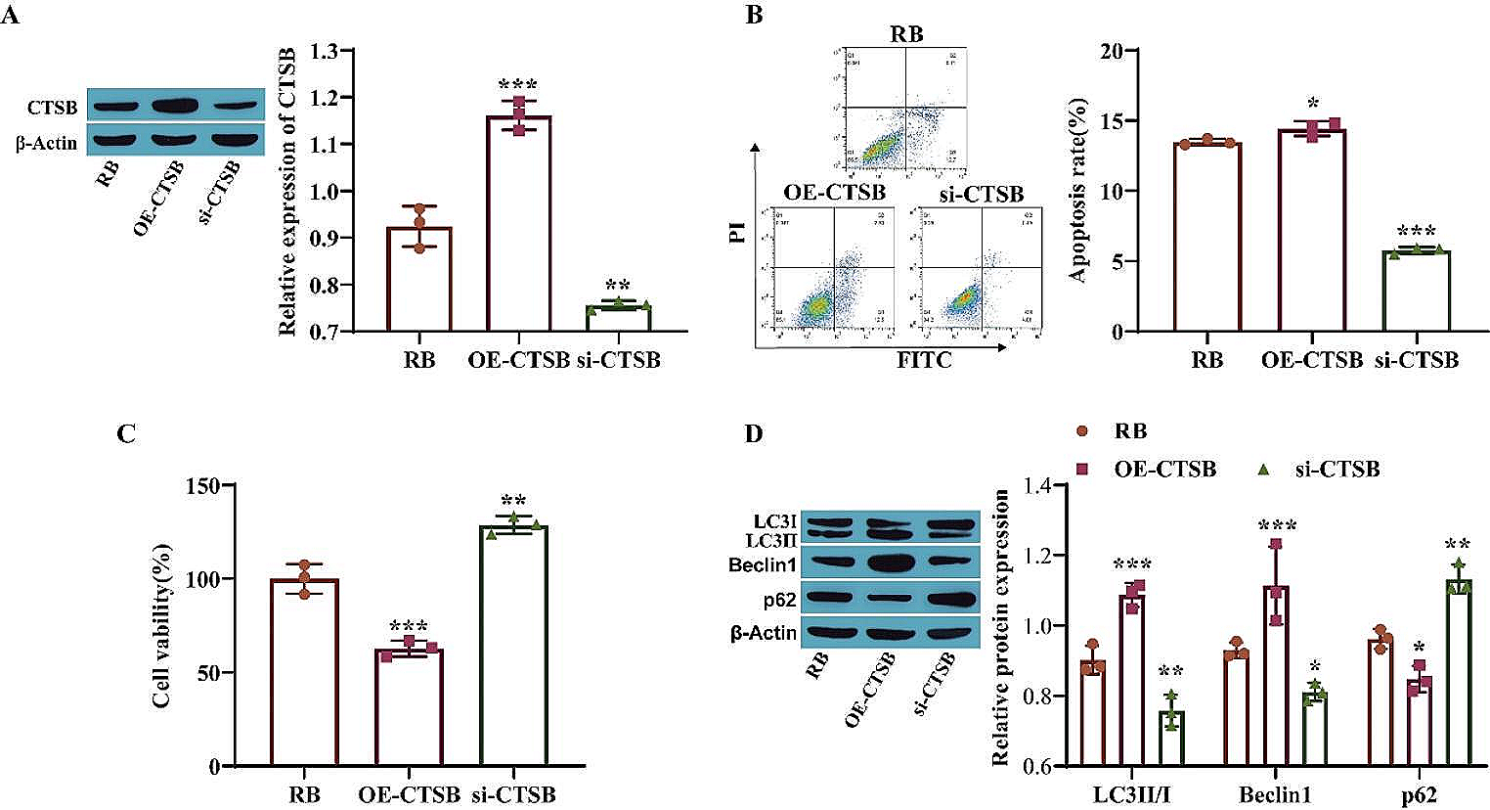
CTSB induces autophagy-dependent death in retinoblastoma cells. **A**: CTSB expression was measured by Western blotting; **B**: Apoptosis was detected by flow cytometry; **C**: Cell viability was measured by a CCK-8 assay; **D**: Western blotting was used to measure autophagy-related protein levels. *P < 0.05, **P < 0.01, ***P < 0.001 vs. the RB group

To examine the effect of CTSB on autophagy-dependent death in RB cells, first, the transfection efficiency of CTSB was examined, and the results showed that, compared with that in the RB group, CTSB expression was significantly upregulated after overexpression of CTSB (OE-CTSB) and significantly downregulated after knockdown of CTSB (si-CTSB) (Fig. 2A), suggesting that the transfection of the OE-CTSB and si-CTSB constructs was successful. Apoptosis was detected by flow cytometry. Compared to that in the RB group, the apoptosis rate was markedly elevated in the OE-CTSB group and markedly decreased in the si-CTSB group (Fig. 2B). Cell proliferation was evaluated by a CCK-8 assay, and the results showed that cell proliferation activity in the OE-CTSB group was significantly lower than that in the RB group, while cell viability was increased after CTSB knockdown (Fig. 2C). In addition, Western blot analysis of autophagy-related protein expression showed that the levels of LC3II/I and Beclin1 were prominently elevated and that the level of p62 was markedly decreased in the OE-CTSB group compared to the RB group, while the opposite trends in terms of changes in protein expression were observed in the si-CTSB group (Fig. 2D). These results suggest that overexpression of CTSB can inhibit the proliferation of Y79 cells and promote apoptosis and autophagy in these cells.

### Lysosomal–Nuclear Translocation of CTSB Promotes DNA Damage

**Fig. 3:**
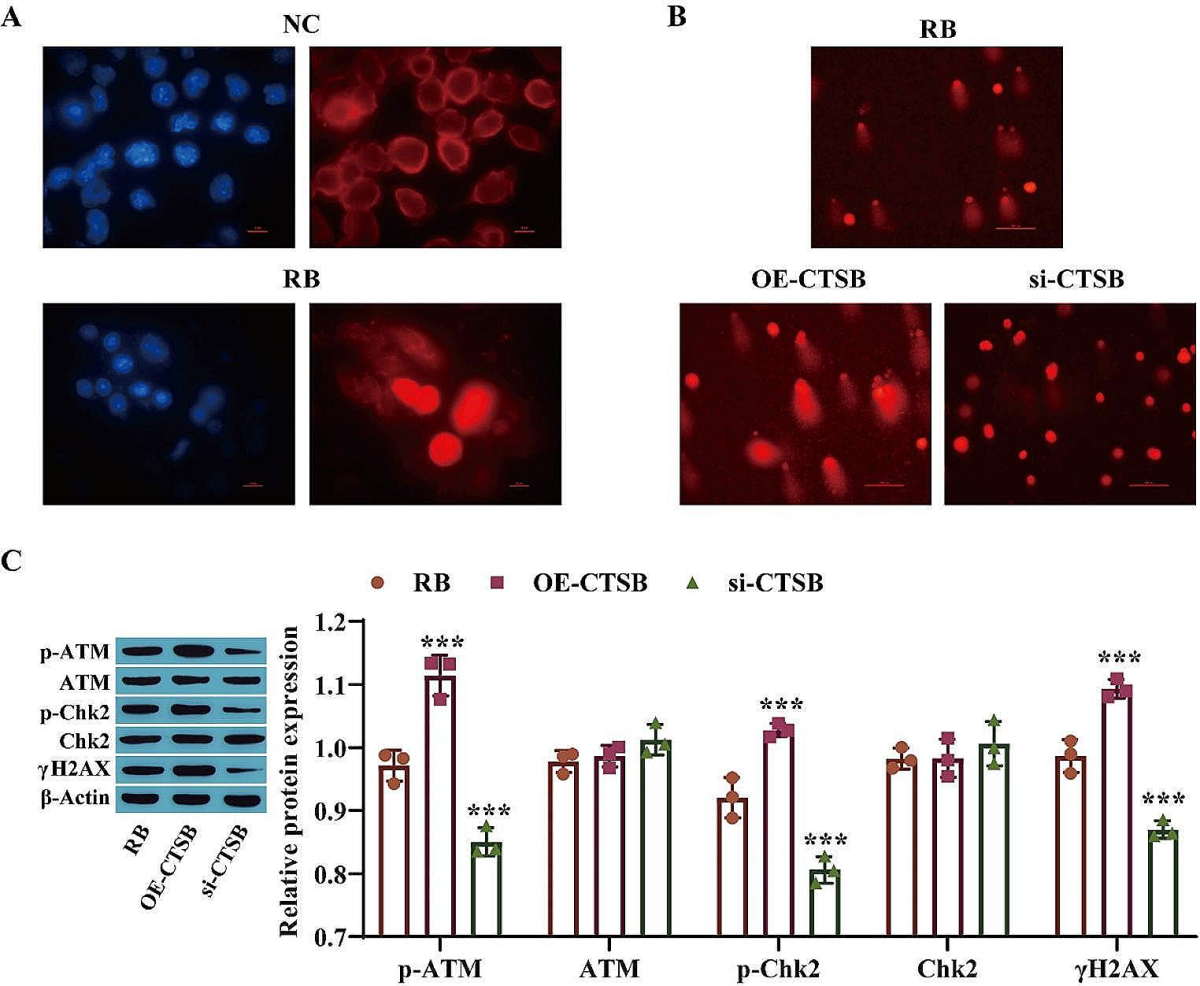
Lysosome–nuclear translocation of CTSB promotes DNA damage. **A**: Nuclear translocation of CTSB was detected by immunofluorescence staining; **B**: DNA damage was detected by a comet assay; **C**: The levels of DNA damage markers were measured by Western blotting. ^***^P < 0.001 vs. the RB group

To determine the effect of the lysosomal–nuclear translocation of CTSB on DNA damage, first, nuclear translocation of CTSB was detected by immunofluorescence staining. The results showed that the CTSB protein was translocated from the cytoplasm to the nucleus in the RB group (Y79 cells) compared with the NC group (ARPE-19 cells) (Fig. 3A). The results of the comet assay showed that overexpression of CTSB promoted DNA damage, while knockdown of CTSB alleviated DNA damage (Fig. 3B). In addition, Western blot analysis of DNA damage markers (ATM, Chk2, and γH2AX) revealed that the levels of p-ATM, p-Chk2 and γH2AX in the OE-CTSB group were markedly higher than those in the RB group, while the levels of these proteins in the si-CTSB group were significantly lower than those in the RB group. However, there was no significant change in the level of ATM or Chk2 (Fig. 3C). These findings suggest that nuclear translocation of CTSB can induce DNA damage in Y79 cells.

### CTSB Induces DNA Damage and Cell Cycle Arrest in Retinoblastoma Cells by Inhibiting BRCA1 Expression

**Fig. 4:**
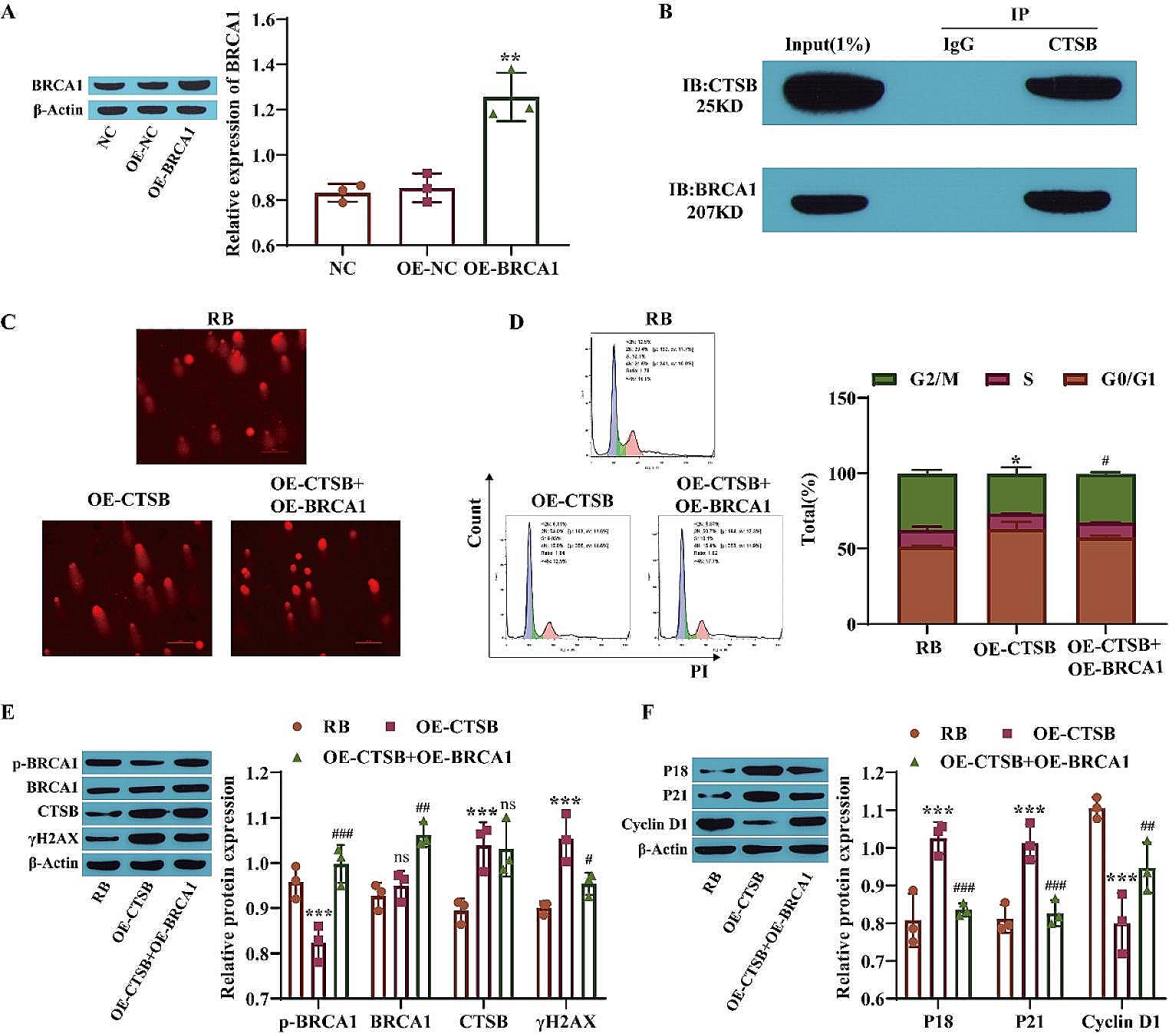
CTSB induces DNA damage and cell cycle arrest in retinoblastoma cells by inhibiting BRCA1. **A**: The expression of BRCA1 was measured by Western blotting; **B**: The interaction between CTSB and BRCA1 was verified by coimmunoprecipitation; **C**: The comet assay was used to measure DNA damage; **D**: The cell cycle in Y79 cells was analyzed by flow cytometry; **E**: Western blotting was used to measure the levels of p-BRCA1, BRCA1, CTSB and γH2AX; F: Cell cycle-related protein levels were determined by Western blot analysis. ^*^P < 0.05, ^**^P < 0.01, ^***^P < 0.001vs. the NC or RB group; ^#^*P* < 0.05, ^##^*P* < 0.01, ^###^*P* < 0.001 vs. the OE-CTSB group

BRCA1 is a tumor suppressor that is present in all cells and is involved in DNA synthesis, contributing to DNA repair and transcriptional regulation in response to DNA damage [30]. Therefore, this study then explored the effect of nuclear translocation of CTSB on DNA damage and cell cycle arrest in RB cells via BRCA1. The overexpression efficiency of BRCA1 was determined by Western blotting. The results showed that, compared with that in the NC group, the expression of BRCA1 in the OE-BRCA1 group was significantly upregulated, indicating successful transfection of OE-BRCA1 (Fig. 4A). The coimmunoprecipitation assay revealed that a large amount of BRCA1 protein was precipitated in the anti-CTSB antibody group, indicating the interaction between CTSB and BRCA1 (Fig. 4B). The results of the comet assay showed that overexpression of CTSB promoted DNA damage, while further overexpression of BRCA1 significantly reduced DNA damage (Fig. 4C). In addition, flow cytometry showed that overexpression of CTSB induced G0/G1 arrest and inhibited cell cycle progression, while overexpression of BRCA1 weakened the influence of CTSB on the cell cycle and promoted cell cycle progression (Fig. 4D). Western blot analysis revealed that the p-BRCA1 level in the OE-CTSB group was markedly lower than that in the RB group, while the levels of CTSB and γH2AX (DNA damage markers) were markedly higher than those in the RB group. However, compared to those in the OE-CTSB group, the levels of p-BRCA1 and BRCA1 in the OE-CTSB + OE-BRCA1 group were increased, while the CTSB level was not significantly changed, and the γH2AX level was clearly decreased (Fig. 4E). Western blot analysis of the expression of cell cycle-related proteins showed that overexpression of CTSB promoted the expression of P18 and P21 and inhibited the expression of Cyclin D1, while overexpression of BRCA1 weakened the effect of CTSB overexpression (Fig. 4F). These results suggest that CTSB promotes DNA damage and cell cycle arrest in Y79 cells by inhibiting BRCA1 phosphorylation.

### CTSB Mediates Lysosomal Stress-Induced Ferroptosis and Autophagy Through the STAT3/STING1 Pathway

**Fig. 5:**
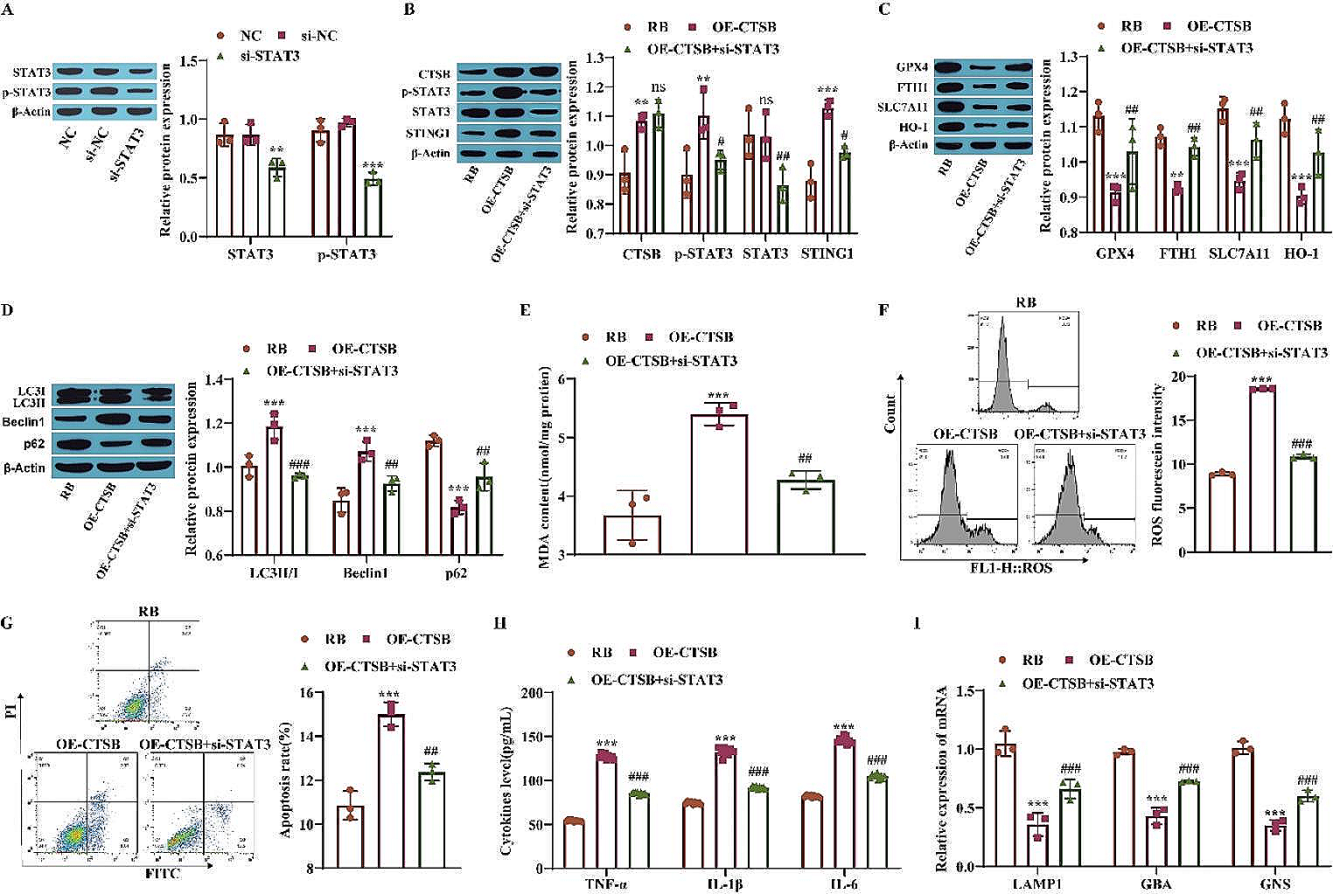
CTSB mediates lysosomal stress-induced ferroptosis and autophagy through the STAT3/STING1 pathway. **A**: The expression of STAT3 was measured by Western blotting; **B**: Western blotting was used to measure the levels of CTSB, p-STAT3, STAT3, and STING1; **C**: Ferroptosis-related protein levels were measured by Western blotting; **D**: Autophagy-related protein levels were measured by Western blotting; **E**: The content of intracellular MDA was measured by a kit; **F**: Flow cytometry was used to measure ROS levels; **G**: Apoptosis was measured by flow cytometry; **H**: The concentrations of inflammatory factors were measured by ELISA; **I**: RT‒qPCR was used to measure the expression of lysosomal markers. ^**^P < 0.01, ^***^P < 0.001 vs. the NC or RB group; ^#^*P* < 0.05, ^##^*P* < 0.01, ^###^*P* < 0.001 vs. the OE-CTSB group

STAT3 is a cytoplasmic transcription factor. The abnormal activation of STAT3 can affect cell growth, survival, angiogenesis and invasion by regulating the expression of certain genes and eventually affect the initiation and progression of tumors [31]. It has also been shown that translocation of CTSB from the lysosome to the nucleus results in STING1-dependent ferroptosis in vitro [32]. Therefore, this study explored the effects of CTSB-mediated lysosomal stress through the STAT3/STING1 pathway on ferroptosis and autophagy. First, the knockdown efficiency of STAT3 was evaluated, and the results showed that STAT3 expression was significantly downregulated in the si-STAT3 group compared with the NC group, indicating that si-STAT3 transfection was successful; moreover, the p-STAT3 level was decreased in the si-STAT3 group (Fig. 5A). Western blot analysis was performed to measure the expression of STAT3/STING1 pathway proteins, autophagy-related proteins and ferroptosis-related proteins. The results showed that overexpression of CTSB increased the protein levels of CTSB, p-STAT3, STING1 and the autophagy-related proteins LC3II/I and Beclin1 and that the expression of the ferroptosis-related proteins GPX4, FTH1, SLC7A11, HO-1 and p62 was inhibited. However, the effects of CTSB overexpression on the expression of various proteins were reversed after STAT3 was knocked down, except that the expression of STAT3 was significantly downregulated and the expression of CTSB was not significantly changed (Fig. 5B-D). Further evaluation of the oxidative stress indices MDA and ROS via a kit and flow cytometry showed that CTSB overexpression promoted intracellular MDA accumulation and ROS accumulation, while STAT3 knockdown weakened the effect of CTSB overexpression and reduced oxidative stress in RB cells (Fig. 5E-F). In addition, the apoptosis rate was significantly reduced after STAT3 knockdown (Fig. 5G). ELISA was used to measure the concentrations of inflammatory factors, and the results showed that CTSB overexpression increased the concentrations of the inflammatory factors TNF-α, IL-1β and IL-6 and that further knockdown of STAT3 reversed the effect of CTSB overexpression (Fig. 5H). The expression of lysosomal markers was measured by RT‒qPCR. The results showed that overexpression of CTSB inhibited the expression of LAMP1, GBA and GNS, while further knockdown of STAT3 promoted the expression of LAMP1, GBA and GNS (Fig. 5I). These findings suggest that CTSB induces lysosomal stress by activating the STAT3/STING1 pathway and promotes ferroptosis and autophagy in Y79 cells.

### CTSB Activation Restrains the Malignant Biological Behavior of Retinoblastoma Cells

**Fig. 6:**
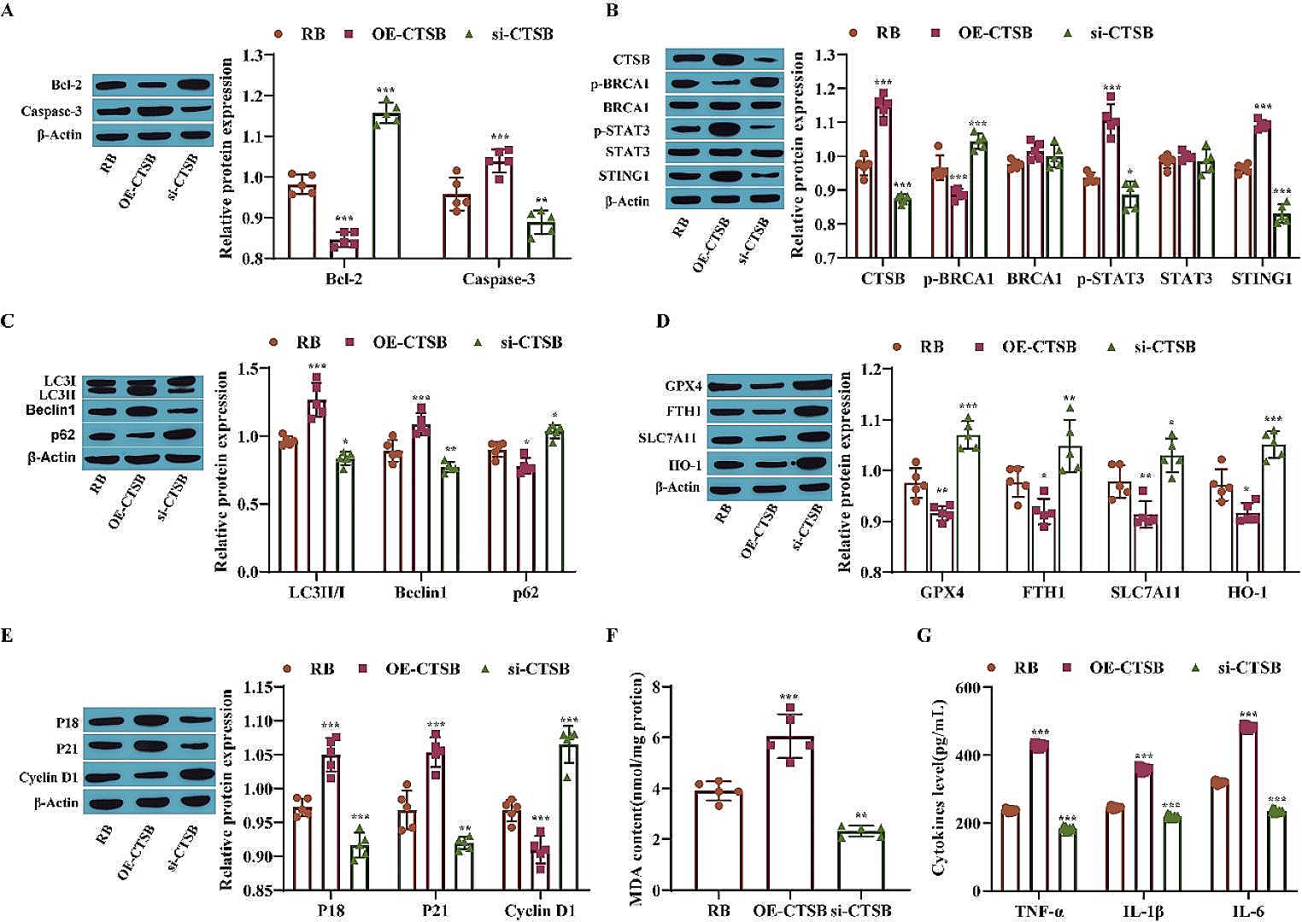
Activation of CTSB restrains the malignant biological behavior of retinoblastoma cells. **A**: The expression of Bcl-2 and Caspase-3 was measured by Western blotting; **B**: The levels of CTSB, p-BRCA1, BRCA1, p-STAT3, STAT3 and STING1 were measured by Western blotting; **C**: The levels of LC3II/I, Beclin1 and p62 were measured by Western blotting; **D**: Western blotting was used to measure the levels of GPX4, FTH1, SLC7A11 and HO-1; **E**: The levels of P18, P21 and Cyclin D1 were measured by Western blotting; **F**: The content of MDA was measured by a kit; **G**: The concentrations of inflammatory factors were assessed by ELISA. ^*^P < 0.05, ^**^P < 0.01, ^***^P < 0.001 vs. the RB group

This study further examined the effect of CTSB activation on the malignant biological behavior of RB cells. Western blotting was used to measure the expression of apoptosis-related proteins, STAT3/STING1 pathway-related proteins, autophagy-related proteins, ferroptosis-related proteins and cell cycle-related proteins. The results showed that the levels of Caspase-3, CTSB, p-STAT3, STING1, LC3II/I, Beclin1, P18 and P21 were significantly elevated in the CTSB overexpression group compared with the RB group, while the levels of Bcl-2, p-BRCA1, p62, GPX4, FTH1, SLC7A11, HO-1 and Cyclin D1 were significantly decreased and the levels of BRCA1 and STAT3 were not noticeably changed. However, the effect of CTSB knockdown on the level of each protein was opposite to that of CTSB overexpression (Fig. 6A-E). In addition, a kit was used to measure the content of MDA, and compared to that in the RB group, the content of MDA was significantly increased after overexpression of CTSB and decreased noticeably after knockdown of CTSB (Fig. 6F). Moreover, the levels of TNF-α, IL-1β and IL-6 were markedly elevated after overexpression of CTSB, and the levels of TNF-α, IL-1β and IL-6 were markedly decreased after knockdown of CTSB (Fig. 6G). The above results show that overexpression of CTSB can effectively promote ferroptosis and autophagy and subsequently inhibit the malignant biological behavior of RB cells.

## Discussion

RB is the most common intraocular malignant tumor in children and is a serious threat to the life, eyes and vision of children [3]. RB is considered a remediable cancer in high-income countries, with disease-free survival rates approaching 100% [33]. Despite some advances in RB treatment, there are still several problems that need to be addressed, including secondary malignancies [34]. Therefore, it is important to search for potential molecular targets for RB treatment. In the present study, GEO database analysis revealed that CTSB was expressed at low levels in RB tissues. Similarly, the results of this study confirmed that CTSB was downregulated in RB animal models and RB cells and that overexpression of CTSB reduced the proliferative activity of Y79 cells and promoted their apoptosis and autophagy. This study further analyzed the molecular mechanism through which CTSB affects the viability of Y79 cells.

Studies have shown that CTSB translocation from lysosomes to the nucleus can induce DNA damage [8]. However, whether nuclear translocation of CTSB can affect DNA damage in RB cells remains unclear. Therefore, in the present study, an immunofluorescence assay was used to determine that CTSB was transferred from lysosomes to the nucleus in Y79 cells, and overexpression of CTSB was found to increase the levels of the DNA damage markers p-ATM, p-Chk2 and γH2AX, which ultimately promoted DNA damage in Y79 cells. However, after knocking down CTSB, DNA damage was inhibited. This finding suggested that nuclear translocation of CTSB can promote DNA damage in RB cells. In addition, BRCA1 has been shown to promote DNA repair and transcriptional regulation in response to DNA damage [30], and Wang [8] reported that RD-N treatment drives CTSB translocation from lysosomes to the nucleus, where it facilitates DNA damage by suppressing BRCA1 expression. In the present study, co-IP was used to confirm the binding between CTSB and BRCA1, and CTSB overexpression was found to inhibit BRCA1 phosphorylation. Further overexpression of BRCA1 inhibited cell cycle arrest in RB cells, accelerated the cell cycle, and alleviated DNA damage, consistent with the findings of previous studies. These results indicate that CTSB nuclear translocation can affect the expression of BRCA1 and participate in RB-related processes.

By further exploring potential biomarkers of RB development, we found that the STAT3/STING1 signaling pathway is involved in RB-related processes. Gao [35] determined that Erastin induces CTSB expression by restoring the expression of STAT3, a transcription factor that promotes lysosomal cell death. Moreover, translocation of CTSB from lysosomes to the nucleus induces STING1-dependent ferroptosis in vitro [32]. Several studies have shown that lysosomes are involved in drug tolerance, cellular adaptation, immune responses, nutrient sensing and cell death under conditions of cellular stress [27]. Joy [36] showed that lysosomal instability and the release of CTSB into the cytoplasm can facilitate apoptosis. In this study, the results showed that nuclear translocation of CTSB can activate the STAT3/STING1 pathway, thereby promoting the expression of autophagy-associated proteins and inflammatory factors, inhibiting the expression of ferroptosis-related proteins and lysosomal markers, and inducing lysosomal stress, thus promoting ferroptosis and autophagy in Y79 cells. However, knocking down STAT3 weakens the function of CTSB. These results suggest that CTSB can affect lysosomal stress through the STAT3/STING1 pathway and subsequently promote ferroptosis and autophagy, thus playing a role in RB.

## Conclusion

This study revealed that nuclear translocation of CTSB can not only promote DNA damage and cell cycle arrest in Y79 cells by inhibiting BRCA1 expression but also alleviate RB by activating the STAT3/STING1 pathway and inducing lysosomal stress, ultimately leading to ferroptosis and autophagy in Y79 cells. These findings suggest that CTSB plays a key role in the development of RB and that CTSB may be an effective target for RB therapy. However, this study has several limitations. This study revealed this phenomenon through only cellular and animal experiments, and clinical investigations are lacking. Clinical samples need to be collected to explore the clinical significance of CTSB in RB in a future study.

## Electronic Supplementary Material

Below is the link to the electronic supplementary material.

Supplementary Material 1

Supplementary Material 2

Supplementary Material 3

Supplementary Material 4

## Data Availability

The datasets used and/or analyzed during the current study are available from the corresponding author upon reasonable request.
