# Supplementary material for: CTSB Nuclear Translocation Facilitates DNA Damage and Lysosomal Stress to Promote Retinoblastoma Cell Death
Source: Mol Biotechnol. 2023 Dec 30;66(9):2583–94. doi: 10.1007/s12033-023-01042-0 (PMC11424708; doi:10.1007/s12033-023-01042-0)
Supplement: Supplementary file 1 — Supplementary Material 1 [file 12033_2023_1042_MOESM1_ESM.docx]

1, CTSB is low expression in RB.

2, CTSB promotes DNA damage by inhibiting BRCA1 phosphorylation in RB.

3, CTSB induces lysosomal stress by activating the STAT3/STING1 pathway and promotes ferroptosis and autophagy in RB cells.
