# Supplementary material for: CTSB Nuclear Translocation Facilitates DNA Damage and Lysosomal Stress to Promote Retinoblastoma Cell Death
Source: Mol Biotechnol. 2023 Dec 30;66(9):2583–94. doi: 10.1007/s12033-023-01042-0 (PMC11424708; doi:10.1007/s12033-023-01042-0)
Supplement: Supplementary file 2 — Supplementary Material 2 [file 12033_2023_1042_MOESM2_ESM.pdf]

Editing Certificate

This document certifies that the manuscript

CTSB nuclear translocation mediates DNA damage repair and lysosomal stress promotes retinoblastoma cell death

prepared by the authors

Cairui Li1#*, Shuguang Sun2#, Yanmei Zhuang3#, Zhaokui Luo4#, Guangquan Ji5, Zhong Liu6

was edited for proper English language, grammar, punctuation, spelling, and overall style by one or more of the highly qualified native English speaking editors at AJE.

This certificate was issued on **December 19, 2023** and may be verified on the AJE website using the verification code **A0E7-068A-AAC7-FCF3-303B**.

Neither the research content nor the authors' intentions were altered in any way during the editing process. Documents receiving this certification should be English-ready for publication; however, the author has the ability to accept or reject our suggestions and changes. To verify the final AJE edited version, please visit our verification page at aje.com/certificate. If you have any questions or concerns about this edited document, please contact AJE at support@aje.com.

AJE provides a range of editing, translation, and manuscript services for researchers and publishers around the world. For more information about our company, services, and partner discounts, please visit aje.com.
