# Supplementary figures and images for: CTSB Nuclear Translocation Facilitates DNA Damage and Lysosomal Stress to Promote Retinoblastoma Cell Death
Source: Mol Biotechnol. 2023 Dec 30;66(9):2583–94. doi: 10.1007/s12033-023-01042-0 (PMC11424708; doi:10.1007/s12033-023-01042-0)

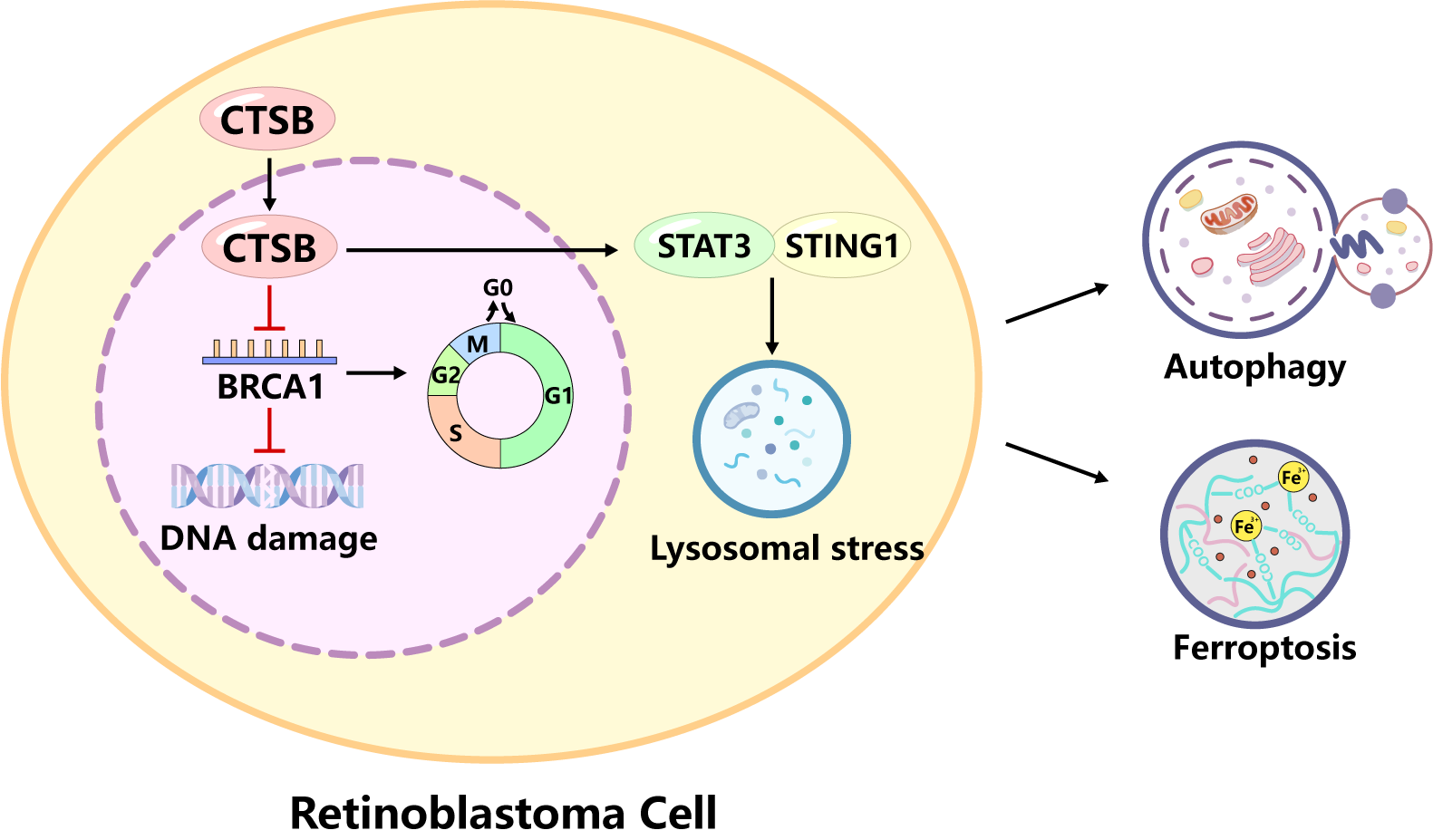

Supplement: Supplementary file 3 — Supplementary Material 3 [file 12033_2023_1042_MOESM3_ESM.tif]
