# Supplementary material for: CTSB Nuclear Translocation Facilitates DNA Damage and Lysosomal Stress to Promote Retinoblastoma Cell Death
Source: Mol Biotechnol. 2023 Dec 30;66(9):2583–94. doi: 10.1007/s12033-023-01042-0 (PMC11424708; doi:10.1007/s12033-023-01042-0)
Supplement: Supplementary file 4 — Supplementary Material 4 [file 12033_2023_1042_MOESM4_ESM.docx]

Dear Editors and Reviewers:

Thank you for your letter and for the editors and reviewer's comments concerning our manuscript entitled "*CTSB nuclear translocation facilitates DNA damage and lysosomal stress to promote retinoblastoma cell death*". These comments are all valuable and very helpful for revising and improving our paper, as well as the important guiding significance to our researches. According to the reviewer's opinion, we have carefully revised the manuscript, which we hope meet with approval. The revised parts of the manuscript have been marked in yellow. The main corrections in the paper and the responds to the reviewer's comments are as follows:

**Responds to the** **reviewer's comments:**

**Reviewer #2:** Manuscript ID: MOBI-D-23-01104
Title: CTSB nuclear translocation mediates DNA damage repair and lysosomal stress promotes retinoblastoma cell death.

Highlight: Not provided.

Graphical Abstract: Not provided.

**Response:** Thank you for your advice. According to your suggestion, we have uploaded the Highlights and Graphical Abstract of the manuscript to the submission system in the form of Supplementary Material.

General Remarks:

The authors have investigated the functionality of CTSB against the RB cell death and found that the CTSB able to induce lysosomal stress, and subsequently relieves RB through cell ferroptosis and autophagy. The study looks interesting but some improvement is required prior to publication.

**Comment 1:** The procedure or the methodology used in the manuscript does not contain any references or citation. The authors should cite the references to provide the validity of the analysis.

**Response:** Thank you very much for your valuable advice. We have supplemented the corresponding references in the manuscript.

**Changes in the paper:** lines 116; lines 143; lines 148; lines 175; lines 182; lines 187; lines 198; lines 228; lines 231.

**Comment 2:** Similar section title is found, e.g. bioinformatics, bioinformatics analysis; the authors can combined both into one section with sub-section stated for date source and analysis. Check and edit throughout the manuscript.

**Response:** Thank you for your advice. Following your suggestion, I have combined both bioinformatics and bioinformatics analysis into one section (2.15).

**Changes in the paper:** lines 239-246.

**Comment 3:** The section title of Section 2.15 has to be edited.

**Response:** Thank you for your advice. We have revised the title of section 2.15 to "Immunohistochemistry analysis". However, in the process of revision, due to minor adjustments in the experimental content of the paper, we deleted the content of immunohistochemistry in section 2.15 without affecting the quality of the paper. Therefore, in the revised version, the title of section 2.15 immunohistochemistry is not presented.

**Comment 4:** Standardized the abbreviation used throughout the manuscript.

**Response:** Thank you for your very helpful advice. We have carefully reviewed the full text, stating the full name and the abbreviation at the first time appears, and using the abbreviation when it appears again.

**Changes in the paper:** lines 8; lines 11-12; lines 19-21; lines 42; lines 52; lines 82-83; lines 93-102; lines 139; lines 145-146; lines 155-168; lines 217-218.

**Comment 5:** Eliminate all the first-person point of view to remain professionalism in journal manuscript.

**Response:** Thank you for your advice. I've eliminated the first-person from the manuscript.

**Comment 6:** Discussion should contain just the discussion on the results obtained and the background / general introduction should be moved to the introduction.
**Response:** Thank you for your very valuable advice. According to your suggestion, we have carefully revised the discussion part of the manuscript.

**Changes in the paper:** lines 436-480.

**Comment 7:** Limitations of the study should be mentioned to provide the research gap for next experiment work.

**Response:** Thank you for your advice. We have pointed out the limitations of this study in the conclusion.

**Changes in the paper:** lines 487-490.

**Reviewer #3:** The results described in the study appear to be interesting, but MAJOR changes are required in the manuscript before it can be reviewed.
**Comment 1:** The readability of the manuscript and understanding/interpretation is extremely difficult because of the choice of words and English usage. It needs to be reviewed thoroughly by a native English speaker. Some examples are Hatcher (Incubator?), impressionable (vulnerable?), momentous (significant), militate (prevent?), hackneyed (ignored?), pernicious (deadly?), fostered (cultured?), deserted (discarded?), embracing (containing?) and so on. The authors should note that the readability of manuscript is significantly enhanced with the use of commonly used (simple) words.
**Response:** Thank you for your very helpful advice. According to your suggestion, we have carefully revised the manuscript and invited professionals to polish the language of the manuscript. The polishing certificate is sent to the submission system in the form of a supplementary document.

**Comment 2:** The hypothesis for the study is not clear/missing in the "Foreword" section.

**Response:** Thank you for your advice. I have explained the hypotheses of this study in the introduction section.

**Changes in the paper:** lines 57-60; lines 70-72; lines 84-90.

**Comment 3:**  Foreword is not the same as Introduction. Please rename the "Foreword" section as "Introduction" since it is an Introduction to the study and not Foreword.

**Response:** Thank you for your advice. I have modified "Foreword" to "Introduction".

**Changes in the paper:** lines 30.

**Comment 4:**  "Foreword": The two most common symptoms of RB are Leukoplakia: Is Leukoplakia a symptom for RB?
**Response:** Thank you for reminding me. Because of our carelessness, we wrote "Leukoplakia" instead of "leukocoria". We have made corresponding modifications.

**Changes in the paper:** lines 32.

**Comment 5:** Many abbreviations are not defined in the manuscript (e.g. RD-N), not defined at the first instance of occurrence (e.g. CTSB), and defined twice (e.g. RB)
**Response:** Thank you for your very helpful advice. We examined the manuscript carefully and defined the abbreviation when it first appeared. According to the requirements of the journal, the abbreviations in the abstract and the text need to be marked with the full name and abbreviation respectively.

**Changes in the paper:** lines 8; lines 11-12; lines 19-21; lines 42; lines 52; lines 82-83; lines 93-102; lines 139; lines 145-146; lines 155-168; lines 217-218.

**Comment 6:** At the end of the Introduction section, what do authors mean with "apply a academic reference for RB clinical remedy"?

**Response:** Thank you for your question. The significance of this study is to provide a potential theoretical basis for the treatment of retinoblastoma. We have made changes to make the description more accurate.

**Changes in the paper:** lines 89-90.

**Comment 7:** The authors should note the difference between the symbols (~) Tilde and (-) hyphen. Tilde indicates equivalency and hyphen is used to indicate range. Throughout the manuscript, the authors have used tilde (~), whereas they should be using hyphen (-).
**Response:** Thank you for your very valuable advice. According to your suggestion, I have made corresponding modifications.

**Changes in the paper:** lines 106; lines 201; lines 203.

**Comment 8:** The authors need to include a Materials section, where details of all the reagents, chemicals, and kits used should be provided. For example, RPMI 1640, DMEM, FBS, BCA kit, Trizol, First strand cDNA synthesis kit, GADPH, D-Hanks solution, CCK-8 reagent, and so on. The details of supplier, city, and country should be mentioned in a consistent way throughout the manuscript.
**Response:** Thank you for your advice. Based on your suggestion, I have supplemented the details of reagents, chemicals and kits to section 2.1(Materials) of the Materials and Methods of the manuscript.

**Changes in the paper:** lines 92-104.

**Comment 9:** The authors should describe the methods in full details so that they can be reproduced.
**Response:** Thank you for your very valuable advice. We have redescribed the experimental methods in the manuscript in detail.

**Changes in the paper:** lines 119-143; lines 148-173; lines 181-196; lines 230-238.

**Comment 10:**  The authors have initiated the sentence with a verb throughout the manuscript. Example: (a) Executed the comet assay….(b) Collected nude mouse retinal…..(c) Harvested Y79 cells…..(d) Deparaffinized paraffin-embedded……(e) Carry out unified background……(f) Obtained thirty male…..(g) Obtained ARPE-19…..(h) Seeded Y79cells…..(i) Determined protein thickness in the light of the BCA assay kit. For scientific writing, the typical sentence structure of subject, verb, and then object is followed.
**Response:** Thank you for your careful review of our manuscript. We examined the manuscript carefully and made the correct changes. In addition, we have invited professionals to polish the language of the manuscript to ensure that the grammar meets the requirements of the journal. Meanwhile, the retouching certificate is also uploaded to the submission system.

**Comment 11:** Cell culture: The authors mentioned "proliferation density reached about 80%": This can be better rephrased as "when cells reached 80% confluency".

**Response:** Thank you for your advice. According to your suggestion, we have made corresponding modifications.

**Changes in the paper:** lines 125.

**Comment 12:** Statistical analysis section: Please correct the whole section. All sentences are either incomplete or framed incorrectly.
**Response:** Thank you for your advice. We have carefully revised the statistical analysis section of the manuscript.

**Changes in the paper:** lines 257-262.

**Comment 13:** Statistical analysis section: The sentence is incomplete or framed incorrectly. "Use of all statistical analysis GraphPad Prism 8.0."
**Response:** Thank you for your advice. We have made corresponding modifications.

**Changes in the paper:** lines 257-258.

**Comment 14:** Statistical analysis section: The sentence is incomplete. "Statistically significant if p<0.05." Suggestion" The statistical significance was established when p-value < 0.05
**Response:** Thank you for your helpful advice. According to your suggestion, we have made corresponding modifications.

**Changes in the paper:** lines 261-262.

**Comment 15:** Statistical analysis section: The sentence is incomplete. "One-way ANOVA and T test as the means of analysis".
**Response:** Thank you for your advice. We have made corresponding modifications.

**Changes in the paper:** lines 259-261.

**Comment 16:**  Results: What do the authors mean with "difference analysis" in the section: The expression of CTSB in retinoblastoma. All the sentences are disconnected from each other. The section needs "synthesis" of results

**Response:** Thank you for your advice. " difference analysis " refers to the application of the GSE97508 dataset for gene differential expression analysis. Meanwhile, we have synthesized the results of Figure 1.

**Changes in the paper:** lines 265-273.

**Comment 17:**  Figure 1A: What is Group A and B?

**Response:** Thank you for your question. I have explained Group A and B in the Figure 1 legend.

**Changes in the paper:** lines 276.
